# A systematic review of the impact of Mental Health First Aid on medical, nursing and allied healthcare professional students

**DOI:** 10.1192/bjo.2026.12062

**Published:** 2026-09-01

**Authors:** Su Ying Yeoh, Sharna Bennett, Helen Bintley, Joanne Rodda

**Affiliations:** Kent and Medway Medical School, Canterbury, UK; Kent and Medway Mental Health NHS Trust, Maidstone, UK

**Keywords:** Mental Health First Aid, MHFA, healthcare professional students, mental health literacy, mental health education

## Abstract

**Background:**

Healthcare professional (HCP) students are at high risk of mental health problems, but stigma and fear of career repercussions often deter them from seeking help. Mental Health First Aid (MHFA) is a globally disseminated course teaching the public to identify and respond to people experiencing mental health problems. MHFA training may address some of the challenges faced by HCP students, by improving mental health knowledge and by enhancing well-being and peer support.

**Aims:**

To systematically review the available literature regarding the impact of MHFA training on HCP students’ mental health literacy, confidence and intentions to provide help, stigma, peer support and self-care.

**Method:**

Following Preferred Reporting Items for Systematic Reviews and Meta-Analyses guidelines (International Prospective Register of Systematic Reviews ID: CRD42024589509), five databases were searched. Primary studies evaluating the above outcome measures in HCP students were included. Two authors independently screened references and extracted data. Quality was assessed using the Modified Medical Education Research Study Quality Instrument and Cochrane Risk of Bias tools. A narrative synthesis was performed.

**Results:**

Of 2367 records screened, 26 met inclusion criteria. Confidence in supporting others and mental health literacy showed the most consistent improvements following MHFA training, whereas evidence for changes in stigma was mixed. Peer support, self-care and student well-being were infrequently examined, although qualitative data suggested that MHFA had improved openness to help-seeking.

**Conclusions:**

MHFA shows promise in enhancing mental health literacy, confidence and intentions, and in reducing stigma, particularly when supplemented with experiential learning. HCP students may benefit from tailoring of such courses to their specific needs, fostering a culture of peer support, enhancing well-being and introducing basic concepts in mental health.

Healthcare professional (HCP) students are at increased risk of developing mental health problems compared with the general population.^1,2^ They face significant academic and clinical demands in addition to those experienced by other students, including long hours, financial pressures and placements far from their social networks, with psychological distress peaking during clinical rotations.^3,4^ These pressures often translate into elevated rates of stress, burnout and depression, particularly among medical students.^5^

Stigma around mental illness and concerns about the impact of disclosure on fitness to practice often deter students from seeking professional help,^6^ and they are more likely to approach peers for support.^7^ HCP students require support themselves, while simultaneously being expected to recognise and respond to mental health difficulties in others during training and in their future clinical practice.

There has also been a growing awareness of burnout, depression and suicide risk among qualified healthcare professionals,^8^ but the extent and nature of formal mental health training within healthcare curricula remain variable, highlighting the potential role of structured interventions. Against this backdrop there has been increasing interest in structured psychoeducational interventions such as Mental Health First Aid (MHFA), which may serve a dual purpose – addressing student well-being and peer support while building core skills for future clinical practice.

## MHFA: background and evidence base

MHFA is a standardised psychoeducational course designed to train members of the public to recognise signs of mental health difficulties, provide first-line practical and compassionate support to others and guide them towards professional help and other sources of support. It also aims to develop self-care skills and destigmatise mental illness. MHFA teaches a five-point action plan with the acronym ‘ALGEE’, for providing first-line support: approach and assess for risk of suicide or self-harm, listen non-judgementally, give reassurance and information, encourage appropriate professional help, encourage self-help.^9^ The course is delivered by an accredited MHFA instructor to groups of up to 30 students, and can be either face-to-face (12–14 h over multiple sessions), online or hybrid.

Evidence from general population studies^10,11^ suggests that MHFA may function as a public health intervention for improving mental health literacy (defined as ‘knowledge and beliefs about mental disorders which aid their recognition, management or prevention’).^12^ MHFA has also been associated with modest improvements in attitudes towards mental illness.^11^ However, HCP students differ from the general population in both training context and responsibilities, occupying a hybrid position between lay person and clinician. The extent to which evidence from general population settings applies to this group therefore remains unclear.

## MHFA training in healthcare professional education

MHFA training has been incorporated into some medical, nursing and pharmacy courses and may increase mental health literacy, equip students to provide appropriate first-line peer support and improve student well-being.^13^ Embedding such training early in the curriculum alongside Basic Life Support has the potential to support parity of esteem, by recognising that both physical and mental health crises require timely, skilled responses.^14^ A recent systematic review including medical, nursing and paramedic students and qualified HCPs reported improvements in several outcomes, including mental health literacy and confidence;^15^ however, its focus on selected professions and mixed trainee–practitioner populations limits the generalisability of findings to the wider HCP student population.

## Research question and objectives

Against this background, this review examines the potential role of MHFA training in the full spectrum of HCP student disciplines. Our research question was: what evidence exists within the published literature regarding the impact of Mental Health First Aid training on healthcare professional students, and what does this evidence suggest about its relevance for healthcare professional education?

The primary objective was to examine the impact of MHFA training on HCP students’ mental health literacy, their perceived confidence and intentions to provide support, stigma, peer support, self-care and student well-being. A further objective was to explore students’ experiences of MHFA and their perceptions of its relevance to their educational and professional context. By synthesising evidence across HCP disciplines and integrating quantitative and qualitative findings, this review aimed to clarify the scope, strengths and limitations of MHFA training in HCP student education, and to identify directions for future research.

## Method

This systematic review was conducted in accordance with the Preferred Reporting Items for Systematic Reviews and Meta-Analysis (PRISMA) Statement. The research protocol was developed prospectively and registered on the International Prospective Register of Systematic Reviews (PROSPERO) on 23 October 2024 (ID: CRD42024589509).

### Search strategy

Searches were performed in collaboration with a research librarian following familiarisation with the literature. Ovid, Medline, PsycINFO, CINAHL and ERIC electronic databases were initially searched on 9 October 2024, and searches were updated on 4 February 2026. Three blocks of search terms were combined using ‘AND’. The first block covered terms related to MHFA, the second covered terms relating to different healthcare professions (e.g. medical, nursing, pharmacy, physiotherapy, etc.) and the third covered terms relating to students and undergraduate education. A relatively large number of keywords were included in the search strategy, to capture the breadth of the different disciplines and recognise multiple synonyms for MHFA in the literature.

Searches were carried out on Google Scholar to capture studies not indexed in the databases. The search terms were entered into Google Scholar, with the three blocks combined using ‘AND’. References were sorted by relevance, with the first 200 screened by title and abstract against database results to identify additional articles, in keeping with recommended guidance for grey literature searches.^16^

The full search strategy and terms are detailed in Supplementary Document 1 available at https://doi.org/10.1192/bjo.2026.12062.

### Study selection

We included peer-reviewed primary research studies published in or translated into English. Inclusion and exclusion criteria are detailed in Table 1. Two authors independently screened articles by title and abstract, with potentially eligible articles reviewed in full. Full papers were assessed against eligibility criteria by the same two authors, with discrepancies resolved through discussion with a third author.

**Table 1:** Inclusion and exclusion criteria for studies Table 1 long description.

Inclusion criteria	Exclusion criteria
Primary study of any design, published in a peer-reviewed journal Studies published in English language or translated into English Delivery of the standard MHFA course Specific data presented regarding HCP students, in any year of study Studies investigating impact of MHFA on one or more of mental health literacy perceived confidence in providing MHFA intentions to provide MHFA attitudes and stigma towards mental illness peer support and student well-being self-care and personal well-being students’ experiences of participating in the course	Non-peer-reviewed studies (e.g. conference abstracts, dissertations) Non-primary studies (e.g. systematic reviews) Studies published in a language that could not be translated into English Delivery of another intervention that is not MHFA, or non-standard MHFA course (e.g. abbreviated MHFA course, psychological first aid)

MHFA, Mental Health First Aid; HCP, healthcare professional.

### Data extraction

Following PROSPERO registration, study-level data were independently extracted starting from 20 November 2024. This was conducted by two authors using a structured data extraction tool, which was used to record study characteristics, outcome domains and key findings across all included articles. Outcome domains were defined deductively in advance and applied during data extraction, informed by the review objectives and aims of MHFA. Discrepancies in extracted data were resolved through discussion with a third author. Data relevant to the review objectives were extracted on setting, study design, participant demographics, intervention (duration, number of sessions, face-to-face or virtual), outcomes evaluated, qualitative responses and study quality using the Modified Medical Education Research Study Quality Instrument (MMERSQI)^17^ and Cochrane Risk of Bias (RoB) II tool^18^ for randomised controlled trials (RCTs).

MMERSQI is a tool used to assess the methodological quality of medical education studies across several domains, including study design, sampling, setting (single- versus multicentre), data analysis and outcomes, with scores ranging from a minimum of 23.5 to a maximum of 100.^17^ Higher scores reflect greater methodological rigour, indicating that robust principles guided the research design, sampling strategies and analytical approaches.

Interrater agreement for MMERSQI was calculated using the intraclass correlation coefficient (ICC). Discrepancies in data extraction between the two authors were resolved in conjunction with a third author.

### Synthesis

A narrative synthesis was undertaken using the data extracted from the included studies, following established guidance for narrative synthesis in systematic reviews.^19^ Studies were grouped by outcome domain (mental health literacy, confidence and intentions to support others, stigma, peer support and self-care), and by study design, to support comparison across the evidence base. Patterns, gaps and differences were examined across studies. The review drew on evidence from both quantitative and qualitative studies. Quantitative findings were summarised using statistical significance and effect sizes where reported. Studies were grouped by outcome domain (e.g. mental health literacy, confidence, stigma) and compared to identify patterns of consistency and divergence across the evidence base. Higher consideration was given to studies with greater methodological rigour, as indicated by MMERSQI. Qualitative findings, including semi-structured interviews and open-ended survey responses, were organised using the same review framework and were used to contextualise and interpret quantitative findings within each domain. This approach is consistent with the guidance for narrative synthesis in systematic reviews outlined by Popay et al,^19^ which informed our methodology throughout.

## Results

### Search results

**Fig. 1:**
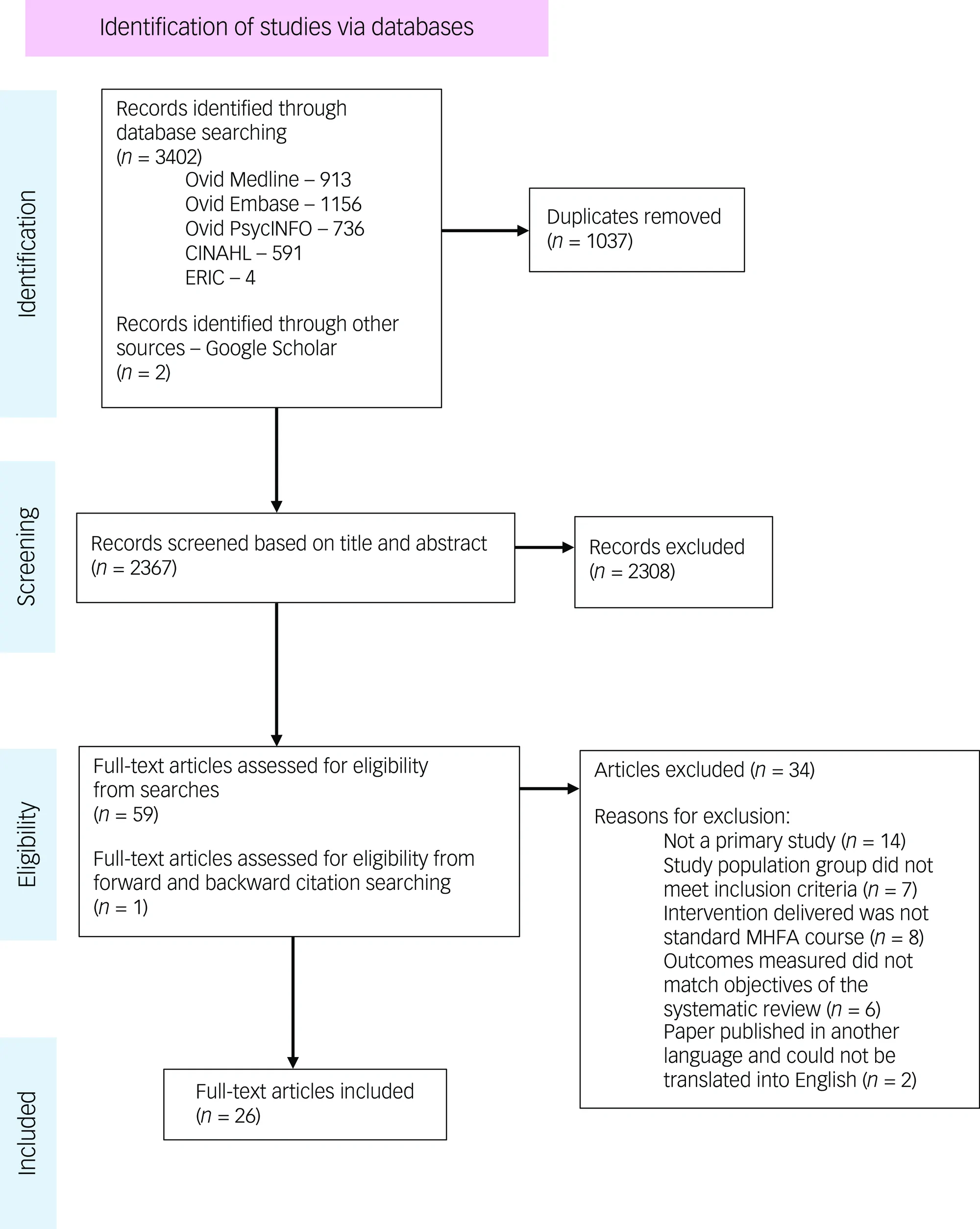
Preferred Reporting Items for Systematic Reviews and Meta-Analysis flow diagram. MHFA, Mental Health First Aid.

The literature search identified 2367 non-duplicated references (Fig. 1), including two additional examples from Google Scholar. One reference was identified through forward citation searching. Sixty full-text articles were assessed for eligibility, of which 26 met eligibility criteria. Reasons for exclusion are detailed in Supplementary Table 1.

### Interrater agreement

Interrater agreement was high for MMERSQI scores (ICC = 0.847), with both reviewers independently in agreement on all overall Cochrane RoB II assessments.

### Study characteristics

Of the 26 studies, 12 were from the USA,^20–31^ 8 from Australia,^32–39^ 2 from Hong Kong^40,41^ and 1 each from the UK,^42^ France,^43^ Canada^44^ and Portugal.^45^ MHFA was translated into a different language in four studies: Portuguese,^45^ Cantonese^40,41^ and French.^43^ Four studies were RCTs.^28,33,40,42^ Three studies were purely qualitative, utilising semi-structured interviews,^37,41,43^ and five quantitative questionnaire studies captured qualitative data through open-ended responses.^21,22,26,34,39^

Fourteen studies included pharmacy students,^20,22–27,30,31,35,36,38^ seven included nursing students,^32,33,40,41,43–45^ three included medical students,^32,42,43^ three included physiotherapy students,^29,34,43^ two included physician assistant students,^21,29^ two included occupational therapy students^28,29^ and one included paramedic students.^39^

MHFA was mostly delivered face to face, with five studies taking a blended approach incorporating elements of virtual and classroom learning^24,28,29,31,39^ and two delivering virtual MHFA.^25,42^ In ten studies, MHFA was embedded into the curriculum^22,24,25,29,31,34,35,37–39^; in all others it was an optional course.

Study characteristics and findings are summarised in Supplementary Table 2. Where reported, effect sizes for statistically significant outcomes were generally small to moderate and are summarised in Supplementary Table 2.

Patterns in area of research focus differed by study design. Quantitative studies predominantly investigated measurable changes in mental health literacy, confidence and intentions to provide help, and stigma. Qualitative studies more broadly explored students’ experiences of MHFA training and their views on its relevance in their education. The distribution of research focus across study designs is summarised in Table 2.

**Table 2:** Distribution of research focus across outcome domains and study designs Table 2 long description.

Area of focus	Quantitative studies	Qualitative studies	Integrated summary
Mental health literacy/knowledge	Dominant	Limited	Core outcome measured quantitatively; there is little qualitative exploration of how the wider impact of this and how this knowledge may be applied in practice
Confidence in supporting others	Dominant	Dominant	Central to both quantitative and qualitative studies
Intentions to provide help	Moderate	Dominant	Frequently measured quantitatively with richer exploration in qualitative studies; translation into behaviour was rarely assessed
Stigma	Moderate	Moderate	Generally a secondary focus across study designs, with consistent but modest focus
Self-care and well-being	Absent	Limited	Limited inclusion across study types; some exploration in qualitative studies
Peer support	Absent	Limited	Mentioned incidentally in qualitative accounts, but not examined systematically or measured quantitatively
Perceived relevance to education and practice	Absent	Moderate	Explored through qualitative accounts, but not measured quantitatively

Categories reflect the relative prominence of outcome domains across the included studies, aligned with the review objectives. Dominant, identified as a primary or central outcome within the majority of studies; moderate, examined repeatedly across studies with variable emphasis; limited, noted descriptively or incidentally without systematic evaluation; absent, not addressed.

### Mental health literacy

Thirteen quantitative studies^20,22,28–30,32,33,36,39,40,42,44,45^ examined the impact of MHFA on mental health literacy.^12^ This was a major area of focus across quantitative studies but a less prominent theme in qualitative studies. Methods of assessing mental health literacy were variable, ranging from multiple-choice questions on MHFA course content,^22,32,42^ responses to case vignettes^33^ and utilisation of the Mental Health Literacy Scale^46^ or an adapted version for HCP students.^29,40^ This methodological variability limits direct comparison across studies, but reflects the breadth of how mental health literacy has been conceptualised within HCP student education.

All quantitative studies except one^22^ reported significant improvements in mental health literacy following MHFA training. One high-quality RCT involving 358 nursing students (scoring 81 out of 100 on MMERSQI) reported significant gains in recognition of mental illness presentations between pre- and post-training,^40^ and another RCT reported that pharmacy students demonstrated greater proficiency in recognising mental illness presentations and supporting evidence-based interventions following training.^36^ These gains were echoed in the qualitative studies, which indicated that students felt that MHFA had meaningfully enhanced their theoretical knowledge of mental illness.^41^

However, the picture was not uniformly positive. One large study of 135 pharmacy students reported no significant differences in knowledge test scores following training,^22^ and another involving medical and nursing students found no improvements in recognition of depression.^32^ The latter finding may be explained by a ceiling effect, with pre-course recognition of depression already at 92% in this sample. Qualitative findings suggested that, given HCP students’ prior mental health knowledge from their training, MHFA content could have been more detailed and challenging.^43^ This convergence between quantitative ceiling effects and qualitative reports of insufficient depth suggests that standard MHFA content may not be sufficiently advanced for HCP student populations, highlighting an opportunity for tailored adaptation of the content for these students. Evidence on the retention of mental health literacy gains was limited but generally positive.

Improvements persisted for up to 6 months post-training for both nursing^40^ and pharmacy students.^31^ Notably, gains were amplified when MHFA was supplemented by additional learning activities; occupational therapy and physician assistant students showed greater gains in mental health literacy when MHFA was supplemented with simulation-based learning.^29^ Additionally, pharmacy students who participated in a community depression screening programme following MHFA demonstrated greater knowledge retention at 8-week follow-up compared with those who received MHFA alone.^30^ This highlights the value of supplementing MHFA with opportunities for applied learning.

### Confidence in providing MHFA

Twenty quantitative studies^20–22,24–26,28,30–36,38–40,42,44,45^ examined the impact of MHFA training on self-rated confidence in providing MHFA. This was a dominant area of focus across both quantitative and qualitative studies. Assessment methods included scoring of self-rated confidence to provide MHFA for a case vignette on a four-point Likert scale,^32,42^ rating confidence in applying subsets of MHFA skills on a five-point Likert scale^21,26^ and through open-ended questions. All studies reported that, following MHFA training, students had significantly increased self-reported confidence in providing support. Similarly, qualitative studies suggested that students had improved communication skills following MHFA training and greater confidence initiating conversations about mental health.^41^ However, this did not apply universally, with some students feeling that they were ‘not ready to intervene’ due to limited practical opportunities in the MHFA course.^43^

Two studies^37,38^ involving pharmacy students described additional increases in self-reported confidence in utilising MHFA skills following classroom learning was reinforced by simulation. Improvements were noted in confidence in asking directly about suicide and encouraging self-help.^38^ Furthermore, pharmacy students who participated in community pharmacy-based depression screening following MHFA training reported significantly higher levels of confidence in applying these skills compared with those who underwent MHFA training alone,^30^ highlighting the value of experiential learning in reinforcing confidence in applying skills.

Interestingly, two studies focusing on skill proficiency following MHFA training suggested that self-rated confidence may not be an accurate reflection of actual skills.^35,38^

In a study by El-Den et al,^35^ 36 pharmacy students participating in simulated scenarios tended to underestimate their ability to approach an individual, and to overestimate their ability to encourage appropriate professional support; although 96% expressed confidence in providing directions for professional support, only 50% actually took the appropriate steps during simulation. Ung et al^38^ also investigated pharmacy students’ skills in simulated patient encounters following MHFA training, finding that students rated their performance lower than tutors and simulated patients. This suggests a disparity between self-rated confidence and assessed performance, and that confidence does not necessarily equate to skill proficiency. Although self-rated confidence in providing support generally improved following training, this did not always translate into skilled responses and some students felt hesitant to intervene following MHFA training.

### Intentions to provide MHFA

There was a moderate focus on the impact of MHFA training on intentions to provide support to others; eight quantitative studies^24,26,27,31–33,40,42^ examined the impact of MHFA on students’ intentions to provide help in real-world situations, and qualitative studies also explored this. All reported that, following MHFA training, students expressed high levels of willingness to provide help, with limited evidence suggesting that this continued for 6 months post-training for pharmacy students.^31^

Furthermore, several studies demonstrated that MHFA training translated into actual helping behaviours, which is promising for an educational intervention; one large RCT involving nursing students reported that, 6 months post-training, 95% in the intervention group (*n* = 86) who encountered an individual in need of mental health support provided help, versus 85% of controls (*n* = 39).^40^ Similarly, 71% (*n* = 72) of paramedic students who had completed MHFA up to 18 months previously reported using skills to support people in their social networks suffering from mental health difficulties, or professionally as first responders.^39^

Willingness to provide support was reflected in the qualitative studies, with MHFA equipping students with the skills and confidence to initiate difficult conversations around mental health that they may previously have avoided. Nursing students described using their training to open conversations about mental illness within their own families, with one participant describing MHFA as a ‘bridge’ between themselves and a parent living with depression,^41^ capturing the role of MHFA in reducing the interpersonal distance that stigma and uncertainty can create. French HCP students also described assisting others with mental health crises following training.^43^ Pharmacy students expressed willingness to provide support despite acknowledging the practical challenges of doing so within the community pharmacy setting,^26^ reflecting a deep sense of responsibility towards others.

Overall, these findings across different HCP disciplines suggest that MHFA may function as more than a knowledge-based intervention, promoting actual helping behaviour across both personal and professional contexts.

### Stigma

There was a moderate focus on the impact of MHFA training on stigmatising attitudes, which was explored in roughly half of the studies. Quantitative studies explored changes in standardised rating scales for stigma, and qualitative studies evaluated shifts in perceptions of individuals with mental illness following MHFA training. Results were conflicting, with ten quantitative studies reporting significant decreases in stigmatising attitudes^23–25,32–34,36,38,40,42^ following training, but four^20,22,28,31^ describing no significant impact. Qualitative studies reported reduced fear and judgement towards people with mental illness following MHFA training, including among nursing students in Hong Kong^41^ and French HCP students, who attributed this to improved understanding of psychotic disorders.^43^

In a study involving delivery of compulsory MHFA to 209 final-year pharmacy students, there was a statistically significant reduction in stigmatising attitudes assessed using the Social Distance Scale immediately post-training. Further reductions in stigmatising attitudes were reported in 86 students who participated in simulation training following MHFA.^38^ Pharmacy students’ self-reported empathy towards individuals with mental illness improved following MHFA training,^22^ as rated by the Kiersma–Chen Empathy Scale.^47^ Although data on the longevity of changes in stigma were limited, nursing students were reported to have further reductions in stigma 2 months post-training compared with immediately afterwards.^33^

Three studies did not report significant shifts in stigmatising attitudes immediately post-training,^20,22,28^ which the authors suggested may reflect positive baseline attitudes and the influence of social desirability bias in answering questions related to stigma. An additional study involving pharmacy students^31^ found that, although there were immediate post-training improvements in stigma, this was not retained at 6-month follow-up. Psychotic illnesses were still perceived as more stigmatising than other mental health conditions following training.^31^

Overall, the evidence suggests that MHFA has potential to reduce stigma among HCP students, particularly if supplemented by experiential learning. However, the variability in findings and limited evidence on the longevity of changes indicate that MHFA as a standalone intervention may not produce lasting shifts in attitudes, especially towards more stigmatising mental health conditions.

### Self-care

No quantitative studies examined the impact of MHFA on students’ self-care as a primary outcome. However, two questionnaire-based studies reported increased willingness to seek help for personal mental health difficulties following training; this included an increase in nursing students’ intentions to seek help for their own mental health (from 71.9 to 91.3%),^45^ and 65 of 235 pharmacy students^24^ reporting proactive help-seeking following MHFA training. One qualitative study provided further insight into self-care-related outcomes among nursing students, who described improved awareness of their own mental health needs and greater willingness to seek support. They also described an impact on resilience, including the use of adaptive coping strategies such as reframing negative situations and communicating more openly with their support networks.^41^

### Peer support and student well-being

This area was not directly addressed in any of the included studies. McGregor et al^44^ assessed nursing students’ self-rated confidence in providing MHFA to a peer experiencing a panic attack before and after a simulated scenario; confidence scores increased significantly following simulation. Qualitative data from semi-structured interviews with French HCPs^43^ suggested that MHFA fostered a sense of kinship within the group, and students proactively offered to support one another if needed in the future. In a further qualitative study, nursing students described being more able to recognise the pressures of their training, and becoming more vigilant for signs of emerging mental health problems in their peers following MHFA training.^41^

### Impact of MHFA in different HCP student disciplines

Although MHFA produces broadly consistent benefits across HCP student populations, limited cross-disciplinary evidence suggests that its impact and relevance vary by student HCP discipline and their anticipated future clinical role.

Pharmacy students were the group studied most frequently^20,22–27,30,31,35–38,43^ and the evidence was consistently positive, with significant improvements reported across mental health knowledge, confidence, intentions to provide support and stigmatising attitudes. Pharmacists have a front-line role, interacting regularly with individuals experiencing mental health difficulties yet typically receiving limited formal mental health training as part of their professional curriculum. The convergence of positive outcomes with a clearly identified professional need makes pharmacy one of the most promising contexts for MHFA implementation identified in this review.

Studies involving medical students similarly reported improvements across multiple outcome domains, including mental health knowledge, confidence, stigma and intentions to provide help. Online and face-to-face courses were similarly effective in this group,^32,42^ with data from one qualitative study suggesting that medical students would value more detailed information about mental disorders.^32^ The combination of positive outcomes but insufficiently advanced content suggests that medical students may benefit from a tailored version of MHFA that better aligns with the depth of clinical knowledge required for their training.

Seven studies^32,33,40,41,43–45^ involving nursing students described similar benefits of MHFA training, with one^44^ reporting increased confidence in supporting a nursing student peer with anxiety and patients with depression following MHFA training. This underscores the dual role of MHFA in helping with professional practice and peer support in this group. Notably, two nursing studies were RCTs with high-quality assessment scores, lending greater methodological weight to the positive findings in this discipline.^33,40^

The evidence base for allied HCP students was considerably thinner, with only one or two studies per discipline for paramedic,^39^ physiotherapy,^29,34^ occupational therapy^28,29^ and physician assistant^21,29^ students. Outcomes were broadly positive, although paramedic students felt that MHFA did not address de-escalation in emergency situations, which would have been of particular relevance to them.^36^ Paramedic students are trained to respond to acute crises, often in high-pressure and unpredictable environments, which MHFA is not designed to address.

Taken together, the cross-disciplinary evidence suggests that the standardised MHFA course may not fully address the profession-specific mental health training needs of students across the full spectrum of healthcare disciplines, and that tailoring of MHFA to different disciplines may be beneficial.

## Discussion

This systematic review synthesises evidence from 26 studies of Mental Health First Aid training for healthcare professional students, spanning 7 countries and a wide range of professional disciplines including pharmacy, nursing, medical, physiotherapy, physician assistant, occupational therapy and paramedic students. Across these contexts, MHFA training was consistently associated with improvements in mental health literacy, self-reported confidence in providing MHFA and intentions to offer support in real-world situations. MHFA simulation appeared to add value when evaluated, by reinforcing knowledge, increasing confidence and demonstrating the gap between perceived and demonstrated skills. Most studies reported reductions in stigmatising attitudes although a minority did not, which may reflect ceiling effects in student populations and social desirability bias. Data on peer support, personal well-being and self-care were more limited, but the available evidence suggests that MHFA may foster mutual support among students and encourage openness to help-seeking. Taken together, these findings suggest that MHFA can be beneficial for HCP students, but also raise the question of whether training tailored specifically to HCP student roles, learning needs and clinical contexts might achieve a more significant and sustained impact.

To our knowledge, this is the first systematic review looking at the impact of MHFA in this group. Our findings are aligned with a recent related review^15^ of 11 studies examining the impact of MHFA in medical, nursing and paramedic students and their qualified counterparts. The authors reported that MHFA significantly increased mental health literacy, confidence and willingness to provide help, reduced stigmatising attitudes and promoted actual helping behaviour among students and qualified HCPs. As an extension of this, our review has a broader scope and, additionally, utilises tools for the assessment of study quality and risk of bias. Overall, the literature base is heterogenous and includes mostly small-scale studies.

In the included studies, MMERSQI scores ranged from 40 to 81 out of 100, indicating considerable variability in methodological quality across the evidence base. Studies scoring in the lower range tended to have single-centre designs, no control group and limited outcome measures, whereas higher-scoring studies employed more robust study designs. This variability is an important consideration when interpreting the review findings.

Most studies lacked long-term follow up, limiting conclusions about whether benefits are sustained over time. Only four RCTs were identified, and three were judged as high risk of bias. Studies assessed as having higher risk of bias reported outcomes broadly consistent with those of other included studies, although interpretation is limited by lack of blinding and control groups.

All included studies were conducted in high-income countries, limiting the generalisability of findings; a pilot study in Uganda^48^ highlighted the challenges of applying Western MHFA curricula in non-Western contexts, and the need for cultural adaptation. Reporting on participant diversity was sparse, despite the relevance of cultural attitudes towards mental illness. Nevertheless, the evidence base comprises 26 studies, capturing both quantitative and qualitative perspectives.

### Mental health literacy

Thirteen quantitative studies reported significant improvements in mental health literacy, with limited evidence that effects may persist for up to 6 months following training.^29,40^ Following MHFA training, students consistently demonstrated improved mental health knowledge and gave more comprehensive responses to case vignettes using the taught framework. This was echoed in qualitative studies, although some students felt that they would have benefitted from more in-depth teaching about the spectrum of mental disorders.^43^

Application of skills appeared to further reinforce these gains, with further improvements in skills following simulated practice^35,38^ and experiential learning by participating in community depression screening.^30^ These findings are consistent with evaluations of MHFA training in wider populations, including the general public, university students and teachers.^11,49,50^ However, a minority of studies found no significant effect;^22,32^ it is possible that this reflects ceiling effects where baseline recognition of common mental health conditions was already high.

Taken together, these findings suggest that MHFA training can support understanding of basic mental health concepts among HCP students, particularly when paired with active learning approaches such as simulation, and that the level of information should be tailored to the students’ prior baseline knowledge. Introducing this training early in undergraduate years may help prepare students for more advanced mental health curriculum teaching and clinical rotations.

### Confidence in providing MHFA

Across 20 studies, MHFA training was consistently associated with increased confidence in providing help, and this was corroborated in qualitative studies. However, several studies noted that there were limited practical opportunities within the scope of MHFA, and simulation was identified as an adjunct for consolidating learning and enhancing confidence by putting skills into practice. Simulation involving individuals with lived experience of mental illness was particularly valued,^38^ reinforcing the human dimension of care. These findings align with wider evaluations of the involvement of individuals with lived experience in healthcare education,^51^ underscoring the importance of holding humanity in mind when delivering clinical teaching. Incorporating such approaches alongside MHFA or similar training may therefore strengthen skill development, improve preparedness for clinical placements and ensure that development of confidence is grounded in practical ability.

### Intentions to provide MHFA

Increased willingness to provide MHFA following training was reported in eight studies and, promisingly, often translated into students taking action in real-world situations.^39,40^ This was supported by qualitative findings; MHFA training equipped students with the confidence, language and communication skills to initiate difficult conversations with friends and family experiencing mental health difficulties, highlighting its potential role in strengthening informal support networks. However, the evidence base remains small, with most studies relying on self-report rather than objective measures, limiting certainty about the extent to which intentions consistently translate into provision of appropriate support. This is particularly relevant given findings from simulation studies showing that students often under- or overestimated their abilities,^35^ suggesting that confidence and willingness to act do not always equate to effective performance.

### Stigma

The impact of MHFA training on stigma was less clear-cut, with ten quantitative studies reporting statistically significant reductions in stigma^23–25,32–34,36,40,42^ but four reporting no meaningful change.^20,22,28,31^ This may be due to high baseline levels of willingness to interact with individuals with mental health problems, as well as potential social desirability in self-reported measures. More fundamentally, stigma is often deeply ingrained^52^ and a single educational intervention such as MHFA training may have limited capacity to shift underlying attitudes. Evidence from an integrative review of the effectiveness of stigma-reducing interventions in ten countries^52^ concluded that contact-based interventions between HCPs and individuals living with mental illness are more effective than educational interventions alone in reducing stigma. This raises the possibility that MHFA could be strengthened by integrating contact with people with lived experience into training, rather than relying solely on information delivery.

### Self-care, peer support and student well-being

Data on peer support and self-care were limited, perhaps unsurprisingly, because these outcomes are challenging to measure objectively. The available quantitative data suggested that students were more receptive to addressing their own mental health.^24,45^ Additionally, qualitative studies suggested that MHFA training increased self-awareness of mental health difficulties and enhanced resilience, supporting students in navigating personal mental health and well-being challenges.^41^ Students were more receptive to providing peer support following training.^43^ Although these findings are of interest, the small evidence base means that the impact of MHFA in this domain remains uncertain and warrants further exploration.

### Perceived relevance to students

Some students reported that MHFA felt too brief or slow-paced, or that it did not cover topics in depth that they considered particularly relevant to their training, including eating disorders, self-harm, gender-based violence,^43^ crisis management in first-response settings^39^ and psychotropic medication counselling.^38^ Although these reflections may suggest areas where greater tailoring could improve alignment with professional training, they may be unrealistic. Based on the MHFA course design, its most valuable contribution is likely to be in building confidence to initiate conversations, promoting openness to self-care and fostering peer support at an early stage of training. The knowledge and skills required for practising HCPs will always require teaching of the relevant course curriculum. Framed in this way, MHFA may complement existing programmes, and future adaptations designed around the needs of different HCP student groups may further strengthen both engagement and impact.

### Impact of MHFA in different student disciplines

Most evidence regarding MHFA in HCP students comes from pharmacy courses, with consistent improvements across multiple outcome domains. Similar benefits are reported in medical and nursing students, supported by some high-quality studies; by contrast, evidence in allied HCP disciplines is sparse. There is limited evidence directly comparing the impact of MHFA across different HCP disciplines, and a stronger evidence base is needed to provide context for tailoring of MHFA courses for different professional groups.

### Strengths and limitations

The strengths of this systematic review include its relatively wide scope compared with other similar reviews, and exploring the impact of MHFA on a broad range of HCP disciplines across multiple outcome measures. Evaluation of qualitative data on students’ experiences of the course is also a strength. The review employed robust methodology and followed PRISMA guidelines. Limitations are closely linked to that of the evidence base, which is relatively small and with many small-scale studies lacking control groups or long-term follow-up. Due to insufficient evidence in the studies identified, it was not possible to clearly address some of the intended aims of the review on the impact of MHFA on peer support, student well-being and self-care. Reporting of discipline-specific outcomes was limited, meaning that the comparative impact of MHFA across student populations could not be determined, and recipient outcomes of individuals receiving support from HCP students were not investigated. A gap in the literature noted during this review is the evaluation of potential unintended negative consequences of MHFA training, which was not explored in any of the included studies; future studies should explicitly assess the potential harms and role boundaries of MHFA and similar interventions in HCP students.

### Future directions

MHFA training is associated with positive outcomes for HCP students, with improvements in mental health literacy, confidence and willingness to provide support and reductions in stigma. Limited evidence suggests that MHFA training encourages HCP students’ openness in seeking help for their own well-being. Simulation and other experiential methods can strengthen these effects by consolidating knowledge, increasing confidence and highlighting gaps between perceived and demonstrated skills.

Embedding MHFA into the curriculum early on may prepare students for clinical rotations, and promote parity of esteem for mental health when delivered alongside established programmes such as Basic Life Support. However, the evidence base remains limited: most studies were small-scale, conducted in high-income settings and lacked long-term follow up, with none evaluating recipient outcomes or unintended negative consequences of MHFA training in this population.

Future research should examine the longevity of training effects, the impact of simulation and adaptation and delivery in low- and middle-income countries. Finally, there is scope to develop bespoke courses tailored to the learning needs, professional responsibilities and well-being challenges of HCP students, which may enhance their ability to support others and maintain their own well-being.

## Supporting information

10.1192/bjo.2026.12062.sm001 Yeoh et al. supplementary material 1 Yeoh et al. supplementary material

10.1192/bjo.2026.12062.sm002 Yeoh et al. supplementary material 2 Yeoh et al. supplementary material

10.1192/bjo.2026.12062.sm003 Yeoh et al. supplementary material 3 Yeoh et al. supplementary material

## Data Availability

Data availability is not applicable to this article because no new data were created or analysed in this study.

## Supplementary material

The supplementary material is available online at https://doi.org/10.1192/bjo.2026.12062

## Table 1 Long description

A table with two columns and eight rows detailing the inclusion and exclusion criteria for studies on mental health first aid (MHFA) courses. The table is divided into two main sections: Inclusion criteria and Exclusion criteria. The Inclusion criteria section lists the following: Primary study of any design, published in a peer-reviewed journal, studies published in English language or translated into English, delivery of the standard MHFA course, specific data presented regarding HCP students in any year of study, and studies investigating the impact of MHFA on one or more of seven specific areas: mental health literacy, perceived confidence in providing MHFA, intentions to provide MHFA, attitudes and stigma towards mental illness, peer support and student well-being, self-care and personal well-being, and students’ experiences of participating in the course. The Exclusion criteria section lists the following: Non-peer-reviewed studies, non-primary studies, studies published in a language that could not be translated into English, delivery of another intervention that is not MHFA, or non-standard MHFA course.

Navigate back to Table 1.

## Table 2 Long description

A table comparing the focus of quantitative and qualitative studies across various areas related to mental health and education. The table has six rows and four columns. The columns are labeled Area of focus, Quantitative studies, Qualitative studies, and Integrated summary. The rows are labeled Mental health literacy/knowledge, Confidence in supporting others, Intentions to provide help, Stigma, Self-care and well-being, Peer support, and Perceived relevance to education and practice. Row 1: Mental health literacy/knowledge, Dominant, Limited, Core outcome measured quantitatively; there is little qualitative exploration of how the wider impact of this and how this knowledge may be applied in practice. Row 2: Confidence in supporting others, Dominant, Dominant, Central to both quantitative and qualitative studies. Row 3: Intentions to provide help, Moderate, Dominant, Frequently measured quantitatively with richer exploration in qualitative studies; translation into behaviour was rarely assessed. Row 4: Stigma, Moderate, Moderate, Generally a secondary focus across study designs, with consistent but modest focus. Row 5: Self-care and well-being, Absent, Limited, Limited inclusion across study types; some exploration in qualitative studies. Row 6: Peer support, Absent, Limited, Mentioned incidentally in qualitative accounts, but not examined systematically or measured quantitatively. Row 7: Perceived relevance to education and practice, Absent, Moderate, Explored through qualitative accounts, but not measured quantitatively.

Navigate back to Table 2.
