# Supplementary material for: A systematic review of the impact of Mental Health First Aid on medical, nursing and allied healthcare professional students
Source: BJPsych Open. 2026 Sep 1;12(5):e222. doi: 10.1192/bjo.2026.12062 (PMC13748678; doi:10.1192/bjo.2026.12062)
Supplement: Supplementary file 1 [file S2056472426120626sup001.docx]

**Supplementary Table 1 – Reasons for Study Exclusion**

Number of articles excluded at the full-text screening stage = 34

| **Reason for exclusion** | **Number of papers** |
| --- | --- |
| Not a primary study | 14 |
| Study population group did not meet inclusion criteria | 7 |
| Intervention delivered was not standard MHFA course | 8 |
| Outcomes measured did not match objectives of the systematic review | 6 |
| Paper published in another language and could not be translated into English | 2 |

List of full-text articles screened and reasons for exclusion:

| **Title** | **Reasons for exclusion** |
| --- | --- |
| Ashoorian D, Albrecht KL, Baxter C, Giftakis E, Clifford R, Greenwell, Barnden J, et al.  **Evaluation of Mental Health First Aid skills in an Australian university population.**  Early Intervention in Psychiatry. 2019 Oct 1;13(5):1121–8. | Study population did not meet inclusion criteria |
| Banh MK, Chaikind J, Robertson HA, Troxel M, Achille J, Egan C, et al.  **Evaluation of Mental Health First Aid USA using the Mental Health Beliefs and Literacy Scale.**  American Journal of Health Promotion. -02-01;33(2):237–47. | Study population did not meet inclusion criteria |
| Bayageldi NK, Kaloglu Binici D.  **Are nursing students ready to respond to disasters? A study on self-efficacy of nursing students to apply psychological first aid.**  Nurse EducToday. 2024 Aug 19;143(Journal Article):106367. | Intervention was not standard MHFA course |
| Carpini, J. A., A. Sharma, M. Kubicki Evans, S. Jumani, E. Boyne, R. Clifford, and D. Ashoorian.  **‘Pharmacists and Mental Health First Aid Training: A Comparative Analysis of Confidence, Mental Health Assistance Behaviours and Perceived Barriers.’** Early Intervention in Psychiatry 17, no. 7 (July 2023): 670–80. | Study population did not meet inclusion criteria |
| Crawford, G., and S. Burns. **‘Confidence and Motivation to Help Those with a Mental Health Problem: Experiences from a Study of Nursing Students Completing Mental Health First Aid (MHFA) Training.’**  BMC Medical Education 20, no. 1 (6 March 2020): 69. | Outcomes measured did not match objectives of the systematic review |
| Crawford, G., S. K. Burns, H. J. Chih, K. Hunt, P. J. M. Tilley, J. Hallett, K. Coleman, and S. Smith.  **‘Mental Health First Aid Training for Nursing Students: A Protocol for a Pragmatic Randomised Controlled Trial in a Large University.’**  BMC Psychiatry 15, no. 1) (pagination (2015): Article Number: 26. Date of Publication: February 19, 2015. | Not a primary study |
| Crespo Gonzalez, C., J. Rae, R. Hill, and S. Dineen Griffin.  **‘Mental Health Training Programmes for Community Pharmacists, Pharmacy Staff and Students: A Systematic Review.’**  Pharmacy Education. Conference: 80th FIP World Congress of Pharmacy and Pharmaceutical Sciences.Seville Spain 23, no. 3 (2023): 62–63. | Not a primary study |
| El-Den S, Chen TF, Moles RJ, O’Reilly C.  Assessing Mental Health First Aid Skills Using Simulated Patients. AmJPharmEduc. Mar;82(2):6222. | Not a primary study |
| ElDen S, Moles RJ, Zhang R, O’reilly CL.  **Simulated patient role-plays with consumers with lived experience of mental illness post-mental health first aid training: Interrater and test re-test reliability of an observed behavioral assessment rubric.**  Pharmacy [Internet]. 2021 Mar 28;9(1) (pp 1-16). | Outcomes did not match objectives of systematic review |
| Eweida, Rasha Salah, Zohour Ibrahim Rashwan, Leena Mohammad Khonji, Abdullah Abdulrahman Bin Shalhoub, and Nashwa Ibrahim.  **‘Psychological First Aid Intervention: Rescue from Psychological Distress and Improving the Pre-Licensure Nursing Students’ Resilience amidst COVID-19 Crisis and Beyond.’** Scienceafrique 19, no. Journal Article (March ): e01472. | Intervention delivered was not standard MHFA course |
| Fessey, Vicki, Gay James, and Theresa Pengelly**.**  **‘Mental Health First Aid: Equipping the Nurses of the Future’.** British Journal of Nursing 25, no. 15 (November 8AD): 858–59. https://doi.org/10.12968/bjon.2016.25.15.858. | Intervention delivered was not standard MHFA course |
| Figueroa, R. A., P. Repetto, and M. Bernales.  **‘Incorporation of Psychological First Aid to the Undergraduate Curriculum.’** Revista Medica de Chile 142, no. 7 (1 July 2014): 934–35. | Not a primary study  Intervention delivered was not standard MHFA course |
| Gapp, D. **‘Implementing Mental Health First Aid and Certification in the Nursing Program.’**  The Journal of Nursing Education 58, no. 6 (1 June 2019): 373. | Outcomes did not match objectives of systematic review |
| Gorton, H. C., H. MacFarlane, R. Edwards, S. Farid, E. Garner, M. Mahroof, S. Rasul, et al.  **‘Mental Health Curricula and Mental Health First Aid in the MPharm.’**  International Journal of Pharmacy Practice. Conference: Health Services Research and Pharmacy Practice Conference, HSRPP 2021.Online 29, no. SUPPL 1 (April 2021): i37–38. | Not a primary study |
| Gorton, H. C., H. Macfarlane, R. Edwards, S. Farid, E. Garner, M. Mahroof, S. Rasul, et al.  **‘UK and Ireland Survey of MPharm Student and Staff Experiences of Mental Health Curricula, with a Focus on Mental Health First Aid.’**  Journal of Pharmaceutical Policy & Practice 14, no. 1 (31 August ): 73. | Not a primary study |
| Habba, S., Stewart, B., & Burghardt, K. (2025). 1138 **From Classroom to Community: Student Pharmacists Utilize Mental Health First Aid Training in a Depression Screening Program.** Journal of the American Pharmacists Association, 65(5). | Not a primary study |
| Jesus Loureiro LM, Santos Rosa AG, Frajuca Pimentel CM, da Cunha SR, da Costa LV, Pereira Correia SI, et al.  **Mental Health First Aid: Contribution of the program to increase mental health literacy in higher education students.** Revista Portuguesa De Investigação Comportamental e Social. -09-01;6(2):24–38. | Paper published in another language and could not be translated into English |
| Kandola, H. S., and S. Minhas.  **‘First Aid for Mental Health: Training for Medical Student Well-Being.’**  Academic Medicine : Journal of the Association of American Medical Colleges 97, no. 3 (1 March 2022): 320. | Not a primary study |
| Kantaris, X., M. Radcliffe, K. Acott, P. Hughes, and M. Chambers.  **‘Training Healthcare Assistants Working in Adult Acute Inpatient Wards in Psychological First Aid: An Implementation and Evaluation Study.’**  Journal of Psychiatric and Mental Health Nursing 27, no. 6 (1 December 2020): 742–51. | Study population did not meet inclusion criteria |
| Kelly, Jenny, and Melanie Birks.  **“It’s the Simple Things You Do First That Start the Process of Help”: Undergraduate Nursing and Midwifery Students’ Experiences of the Mental Health First Aid Course’.**  Collegian 24, no. 3 (6AD): 275–80. https://doi.org/10.1016/j.colegn.2016.04.004. | Intervention delivered was not standard MHFA course |
| Kitchener, Betty A., and Anthony F. Jorm.  **‘The Role of Mental Health First Aid Training in Nursing Education: A Response to Happell, Wilson & McNamara (2015)’.**  Collegian 24, no. 3 (6AD): 313–15. https://doi.org/10.1016/j.colegn.2016.06.002. | Not a primary study |
| Lo, Kristin, Geoffrey Ahern, Alyssia Rossetto, and Melanie K. Farlie.  **‘Mental Health First Aid Training for Allied Health Clinical Educators: A Pre- and Post-Evaluation’.**  Physiotherapy Canada 76, no. 1 (2AD): 55–63. https://doi.org/10.3138/ptc-2021-0099. | Study population did not meet inclusion criteria |
| Maslo, T., J. Robinson, D. BrandEubanks, and A. Kim. **‘Expanding the Pharmacists’ Skill Set: Mental Health First Aid Training.’**  Journal of the American Pharmacists Association.Conference: 2018 Annual Meeting of the American Pharmacists Association, APHA 2018.Nashville, TN United States 58, no. 3 (June 2018): e99. | Not a primary study |
| McCabe, O. L., Charlene Perry, Melissa Azur, Henry G. Taylor, Mark Bailey, and Jonathan M. Links.  **‘Psychological First-Aid Training for Paraprofessionals: A Systems-Based Model for Enhancing Capacity of Rural Emergency Responses’**.  Prehospital & Disaster Medicine 26, no. 4 (2011): 251–58. https://doi.org/10.1017/S1049023X11006297. | Study population did not meet inclusion criteria  Intervention delivered was not standard MHFA course |
| McCann, T. V., and E. Clark.  **‘Australian Bachelor of Midwifery Students’ Mental Health Literacy: An Exploratory Study’.**  Nursing & Health Sciences 12, no. 1 (3AD): 14–20. https://doi.org/10.1111/j.1442-2018.2009.00477.x. | Intervention delivered was not standard MHFA course |
| Nguyen W, Moles R, O’Reilly C, Robinson J, Brand-Eubanks D, Kim A, et al.  **Observed behaviours and suicide assessment language post-Mental Health First Aid training in Australia and the United States: a mixed methods study using discourse analysis**. BMC Medical Education. Dec;22(1):838. | Outcomes did not match objectives of systematic review |
| Pham, L., R. J. Moles, C. L. O’Reilly, M. J. Carrillo, and S. ElDen.  ‘**Mental Health First Aid Training and Assessment in Australian Medical, Nursing and Pharmacy Curricula: A National Perspective Using Content Analysis.**’  BMC Medical Education 22, no. 1 (29 January 2022): 70. | Outcomes did not match objectives of systematic review  Intervention delivered was not standard MHFA course |
| Rodgers, Gina, Sharyn Burns, and Gemma Crawford.  **‘“I Was Able to Actually Do Something Useful”: Evaluating the Experiences of University Students after Completing Mental Health First Aid: A Mixed-Methods Study’.**  Advances in Mental Health 19, no. 1 (-03-01): 40–62. https://doi.org/10.1080/18387357.2019.1663709. | Study population did not meet inclusion criteria |
| Sherwood, D. A., A. Chace, and W. Sargent.  **‘Addressing Pharmacy Student Stigma toward Mental Illness: A Psychiatric Pharmacy Elective Course Including Mental Health First Aid USA Certification.’**  Journal of Pharmacy Practice.Conference: 19th Annual Meeting of the College of Psychiatric and Neurologic Pharmacists, CPNP.Colorado Springs, CO United States 29, no. 3 (June 2016): 290–91. | Not a primary study |
| Zemel, R. ‘**A COVID-19 Call to Action: Psychological First Aid Training for Medical Professionals and Trainees.’** Academic Psychiatry : The Journal of the American Association of Directors of Psychiatric Residency Training and the Association for Academic Psychiatry 46, no. 6 (1 December 2022): 769–73. | Not a primary study |
| New papers found from re-run of searches on 3/4/25: |  |
| Ung, Tina X., et al. **"Simulated psychosis care role-plays for pharmacy curricula: a qualitative exploration of student experiences**." Social Psychiatry and Psychiatric Epidemiology 59.7 (2024): 1269-1277. | Outcomes did not meet objectives of systematic review |
| Vieux, Maxime, Emmanuel Poulet, and Édouard Leaune. "Effets d’une formation de type premiers secours en santé mentale sur les attitudes des étudiants en médecine: une étude mixte contrôlée non randomisée." Annales Médico-psychologiques, revue psychiatrique. Elsevier Masson, 2025.  **Translated title: Effects of a mental health first aid training on the attitudes of medical students: A mixed, non-randomized controlled study** | Paper published in another language and could not be translated into English |
| Advincula, Trisha Joy, Mindraani Abubakar, and Rogie Royce Carandang. **"Effects of Mental Health First Aid Training in Hospital and Community Pharmacists: A Systematic Review." Journal of the American Pharmacists Association (2025).** | Not a primary study |
| Li, Li, et al. **"Mental health first aid training and assessment for healthcare professionals and medical nursing students: a systematic review**." BMC psychology 13.1 (2025): 186. | Not a primary study |
