# Supplementary material for: A systematic review of the impact of Mental Health First Aid on medical, nursing and allied healthcare professional students
Source: BJPsych Open. 2026 Sep 1;12(5):e222. doi: 10.1192/bjo.2026.12062 (PMC13748678; doi:10.1192/bjo.2026.12062)
Supplement: Supplementary file 2 [file S2056472426120626sup002.docx]

| **sStudy** | **Study population** | **Country** | **Study design** | **Objectives of study** | **Relevant outcomes** | **Programme** | **Duration (hours)** | **Mode of delivery** | **MHFA compulsory or voluntary** | **% female** | **Mean age, SD, range** | **Comparison condition** | **Follow up duration** | **Results** | **MMERSQI / ROBII** |
| --- | --- | --- | --- | --- | --- | --- | --- | --- | --- | --- | --- | --- | --- | --- | --- |
| **Medical students** | | | | | | | | | | | | | | | |
| **Davies et al. 2016**  [42] | Medical students (intervention n=27, control n=28)  First, second or third year of study | UK | Quantitative study  RCT | To evaluate the impact of a self-directed MHFA e-learning course on mental health literacy, confidence in providing help, intentions and stigmatising attitudes | Mental health literacy  Confidence  Intentions  Stigma | MHFA online course | Expected time to complete 6-8 hours | Online | Voluntary | 65.6 | 19.9 (3.2), 18-39 | Wait-list control group | Immediately after | The MHFA e-learning group had statistically significant increases in mental health knowledge (p=0.03) confidence (p<0.001), intentions (p<0.001) and decreased stigma (p=0.04) compared to the control group.  The intervention group was more likely to report that they had actively used MHFA skills to support others in real life (p=0.006). | 68  High risk of bias |
| **Nursing students** | | | | | | | | | | | | | | | |
| **Burns et al. 2017**  [33] | Nursing students (intervention n=92, control n=89)  First year of study | Australia | Quantitative study  RCT | To investigate the impact of MHFA training on MHFA intentions, mental health literacy, confidence in providing help and stigmatising attitudes | Mental health literacy  Confidence  Intentions  Stigma | Tailored MHFA | 13 | Face to face | Voluntary | 83.6 | Not stated | Wait-list control group | Immediately after, 2 months | MHFA was associated with statistically significant increases in the intervention compared to control group, in mental health knowledge (p<0.001), confidence (p<0.001), intentions (p<0.001) and decreased stigma (p<0.05) across all three time points.  Stigma continued to decrease at each time point, and confidence continued to increase for the intervention group with the most positive results 2 months post course. | 69  High risk of bias |
| **De Jesus Loureiro et al.** 2019  [45] | Nursing students (n=46)  Second, third or fourth year of study | Portugal | Quantitative study  Uncontrolled pre-and post-test design | To evaluate the impact of MHFA training on mental health literacy, confidence to provide help and intentions to seek help for themselves if required. | Mental health literacy  Confidence  Self-care | MHFA translated into Portuguese | 14 | Face to face | Voluntary | 87.5 | 20.74 (2.2), 19-31 | None | Immediately after | After MHFA training, there were statistically significant improvements in mental health knowledge (p<0.05), confidence to provide help (p<0.01) and intentions to seek help for themselves if needed (p<0.01). | 62.5 |
| **Hung et al. 2019**  [41] | Nursing students (n=25)  All years of study | Hong Kong | Qualitative study  Semi-structured interviews and thematic analysis of content | To explore nursing students’ perceptions and experiences of participating in MHFA training | Qualitative experiences of participating in the course | Standard MHFA course | 12 | Face to face | Voluntary | Not stated | Not stated | None | Immediately after | Five overarching themes regarding the participants’ experiences of the MHFA training were identified: (1) reinforced knowledge and understanding, (2) enhanced techniques and skills, (3) rectified beliefs and values, (4) improved self-awareness of personal mental health status and (5) a sense of achievement. | 40 |
| **Hung et al. 2020**  [40] | Nursing students  (intervention n=182, control n=176)  All years of study | Hong Kong | Quantitative study  RCT with pre-test, post-test and follow up design | To evaluate the impact of MHFA on mental health literacy, confidence and intentions to provide MHFA, stigma and self-care | Mental health literacy  Confidence  Intentions  Stigma | Standard MHFA course | 12 | Face to face | Voluntary | 80.1 | 20.8 +/- 1.91 | Wait-list control group | Immediately after, 6 months | MHFA training was associated with statistically significant increases in mental health literacy (p<0.01) including recognition of depression and schizophrenia, confidence (p<0.01), social distance (p<0.01), and Chinese Health questionnaire score measuring personal wellbeing (p<0.01) compared to the control group. The effectiveness was sustained at 6 month follow up for all the measures except personal wellbeing. 45% of participants in the experimental group reported that they had delivered MHFA following training. | 81  Low risk of bias |
| **McGregor et al. 2023**  [44] | Nursing students (n=22)  First or second year of study | Canada | Quantitative study  Uncontrolled pre-and post-test design | To investigate the impact of MHFA training on nursing students’ confidence to provide support to a peer, and in a clinical scenario. | Confidence | Youth MHFA | 14 | Face to face | Voluntary | Not stated | Not stated | None | Immediately after | Following MHFA training, nursing students reported increased levels of confidence to provide MHFA to a nursing peer struggling with anxiety (p<0.001) and a patient presenting with suicidal ideation (p<0.001), which were statistically significant. | 53.5 |
| **Pharmacy students** | | | | | | | | | | | | | | | |
| **Dulellari et al. 2022**  [20] | Pharmacy students (n=55)  All years of study | USA | Quantitative study  Uncontrolled post-intervention survey | To assess the impact of MHFA on mental health knowledge, confidence, stigmatising attitudes, and appropriateness of the MHFA course for pharmacy students | Mental health literacy  Confidence  Stigma | Standard MHFA course | Not stated | Face to face | Voluntary | 83 | 24.5 (21-33), SD not stated | None | Immediately after | Following MHFA training, there were statistically significantly increases in mental health knowledge (p<0.05) and confidence to provide help (p<0.001). No significant changes reported in stigma. 55% of students felt that the pace and length of the course was not appropriate and 25% expressed that they would have preferred a MHFA course tailored to pharmacy students. | 49 |
| **El-Den et al. 2018**  [35] | Pharmacy students (n=36)  Fourth year of study | Australia | Quantitative study  Uncontrolled post-test design | To evaluate MHFA skills using simulated patients and to compare self-reported confidence in providing MHFA with assessed performance during simulated patient roleplays. | Mental health literacy  Confidence | Standard MHFA course | Not stated | Face to face | Compulsory | Not stated | Not stated | None | Immediately after | Students’ self-reported confidence to apply different MHFA skills did not fully match assessed performance. Students frequently avoided using suicide-specific terminology in assessments (71% in postnatal depression roleplay and 40% in the suicide roleplay). | 57 |
| **Frick et al. 2021**  [22] | Pharmacy students (n=135)  Third year of study | USA | Quantitative questionnaire study supplemented by qualitative free-text analysis  Uncontrolled pre-and post-test design | To assess the impact of MHFA on mental health knowledge, attitudes, self-efficacy and empathy towards people with mental health problems. | Mental health literacy  Confidence  Stigma | Standard MHFA course | 8 | Face to face | Compulsory | 66 | 25.1 (22-35, SD not stated) | None | Immediately after | MHFA training was associated with statistically significantly improvements in confidence (p<0.001) and empathy (p=0.01). There were no statistically significant differences reported for mental health knowledge (p=0.06) or social distance (p=0.2). | 62 |
| **Habba et al.**  **2025**  [30] | Pharmacy students (n=20)  Second to fourth year of study | USA | Quantitative study  Uncontrolled pre- and post-test design | To assess student pharmacist confidence and knowledge in navigating mental health conversations after MHFA alone, versus MHFA paired with experiential learning opportunities through community pharmacy-based depression screening events | Mental health literacy  Confidence | Standard MHFA course | 8 | Face to face | Voluntary | Not stated | Not stated | None | 8 weeks | Students who completed both MHFA training and depression screening events showed greater improvement in mental health knowledge (p=0.03) and confidence (p=0.03) compared to those who completed MHFA training alone. At 8 week follow up, Student pharmacists who participated in both MHFA training and a depression screening event had a significant increase in mental health knowledge compared to those who completed MHFA training alone. Interestingly, there was a significant 11.6% decrease in post-intervention knowledge scores for the group who did not participate in screening at 8 week follow up, suggesting that there was difficulty retaining information if not put into practice. | 57 |
| **McCormack et al. 2018**  [23] | Pharmacy students (n=34) and non-pharmacy students (n=21)  All years of study | USA | Quantitative study  Uncontrolled pre-and post-test survey design | To investigate the effect of MHFA training on stigmatising attitudes towards mental illness among pharmacy and non-pharmacy students | Stigma | Standard MHFA course | 8 | Face to face | Voluntary | 76 | Not stated | None | Immediately after | MHFA was effective in decreasing stigmatising attitudes in pharmacy students. There was a statistically significant improvement in social distance scores post-training (p<0.05). Pharmacy students demonstrated greater reductions in stigma post-training compared to non-pharmacy students. Levels of stigma against schizophrenia remained higher than for depression. | 56.5 |
| **McKeirnan et al. 2023**  [24] | Pharmacy students (n=235)  First, second or third year of study | USA | Quantitative study  Uncontrolled pre-and post-test design | To investigate the impact of MHFA training on pharmacy students’ confidence, intentions to provide MHFA and attitudes towards mental illness. | Confidence  Intentions  Stigma | MHFA (blended course) | 7.5 | 2 hours pre-work and 5.5 hour face to face session | Compulsory | Gender distribution presented in 3 different year groups (ranged from 57.8 – 70.3% female, with no non-binary students | Age presented in 3 different year groups (mean age per year group ranged from 23.8 – 25.6) | None | Immediately after | MHFA training was associated with statistically significant increases in confidence to provide MHFA (p<0.001), intentions to provide help (p<0.001) and statistically significant decreases in 9 out of 15 items on the Opening Minds to Stigma scale.  32% of students reported deciding to proactively seek help for their own mental health. | 55 |
| **McKeirnan et al. 2025**  [31] | Pharmacy students (n=235) | USA | Quantitative study  Uncontrolled post test design | To investigate the longitudinal impact of MHFA training after 6 months, on pharmacy students’ confidence, intentions to provide MHFA and attitudes towards mental illness. | Confidence  Intentions  Stigma | MHFA (blended course) | 7.5 | 2 hours pre-work and 5.5 hour face to face session | Compulsory | Not stated | Not stated | None | 6 months | 6 months post MHFA training, students maintained improvements in overall confidence (p<0.01) and willingness (p<0.01) to provide help. However, a reduction in stigma was only maintained in a few areas. Promisingly, MHFA training prompted 29% of students (n=44) to recognise their own mental health needs and to seek support for themselves. | 53 |
| **O’Reilly et al. 2011**  [36] | Pharmacy students (n=60)  Third year of study | Australia | Quantitative study  Non-randomised controlled design | To investigate the impact of MHFA training on pharmacy students’ mental health literacy, confidence in medication counselling and stigma towards mental illness 6 weeks after course. | Mental health literacy  Confidence  Stigma | MHFA | 12 | Face to face | Voluntary | 64 | 21 (1.99, 19 – 35) | None | 6 weeks | Following training, pharmacy students were significantly more confident in providing pharmaceutical services including medication counselling to patients with mental illness (p<0.01). There were statistically significant reductions in social distance scores (p<0.001) and increased mental health literacy (p=0.004). | 71 |
| **O’Reilly et al. 2019**  [37] | Pharmacy students (n=22)  Final year of study | Australia | Qualitative study  Semi-structured interviews and thematic analysis of content | To use a novel MHFA assessment approach involving simulated roleplays enacted by people with lived experience of mental illness, and explore MHFA participants’ and simulated patients’ views of participating in simulated roleplays. | Qualitative experiences of participating in the course | MHFA | 12 | Face to face | Compulsory | Not stated | Not stated | None | 3 months | Five themes emerged including: the benefits of participating; the value of having a lived experience; challenges with suicide assessment; communication; and the value of immediate feedback and debrief. | 43 |
| **Ung et al. 2024**  [38] | Pharmacy students (n=86)  Final year of study | Australia | Quantitative study  Uncontrolled pre-and post-test design | To explore the impact of MHFA training and simulated psychosis roleplay scenarios on pharmacy students’ confidence, stigma and behaviours when supporting people with mental health problems. | Mental health literacy  Confidence  Stigma | MHFA | 2 days | Face to face | MHFA was compulsory, participation in roleplay was voluntary | 71 | Mean 24.4 (SD 4.6) | None | Unclear | MHFA was associated with statistically significant improvements in confidence to provide help (p<0.05). There was also a statistically significant reduction in stigma following training (p<0.05), which reduced further after students engaged with simulation (p<0.05). Overall, there was significant improvement in 14 out of 15 survey items post-MHFA. For the role plays, students' self-assessment mean scores were the lowest and the MHCEs’ (Mental health consumer educator) mean scores highest. Average scores were lowest for the scenario involving psychosis and suicidal ideation, and highest for the scenario on medication counselling. | 69 |
| **Vickery et al. 2023**  [25] | Pharmacy students (n=69)  First year | USA | Quantitative study  Uncontrolled pre and post-test design | To investigate the impact of MHFA training on pharmacy students’ confidence to provide help and stigmatising attitudes. | Confidence  Stigma | Virtual MHFA | 8 hours | Virtual | Compulsory | 71 | Not stated | None | Immediately after | After MHFA training, there was a statistically significant increase in recognition of stigmatising language associated with mental illness and on all stigma items (p<0.001). There was also a statistically significant improvement in confidence to provide help (p<0.001). | 49 |
| **Witry et al. 2020**  [27] | Pharmacy students (n=23), qualified pharmacists (n=45), other related pharmacy professionals (n=28) | USA | Quantitative study  Uncontrolled post-test design | To measure pharmacy professionals’ preparedness to utilise skills gained from MHFA training | Intentions | MHFA | Variable, 8 hour session or two 4 hour sessions | Face to face | Voluntary | 62 | Range <25 to > 55 | None | Immediately after | After MHFA training, participants reported high levels of preparedness to perform subsets of MHFA skills. They reported greater preparedness to provide MHFA to patients with depression and anxiety than psychosis, eating disorders or substance misuse. | 45 |
| **Witry et al. 2020**  [26] | Pharmacy students (n=23), qualified pharmacists (n=45), other related pharmacy professionals (n=28) | USA | Quantitative questionnaire study supplemented by qualitative free-text analysis  Uncontrolled post-test design | To describe pharmacy professionals' confidence and reluctance to intervene in mental health crises, describe their self-reported use of MHFA behaviours after training, and describe participant open-ended feedback on the course. | Confidence  Intentions  Qualitative experiences of participating in the course | MHFA | Variable, 8 hour session or two 4 hour sessions | Face to face | Voluntary | 62 | Range <25 to > 55 | None | Immediately after | Following MHFA training, participants reported overall high levels of confidence in performing a range of MHFA skills, and low levels of reluctance to provide assistance. The majority of participants (61%) had referred someone to resources due to a mental health crisis, and 44% had asked about suicidal ideation. Open-ended responses indicated that MHFA training was a positive experience, and recommendations for additional training focussed on the pharmacy setting. Time pressures and lack of privacy in the pharmacy setting were cited as challenges in delivering MHFA in pharmacy settings. | 55 |
| **Allied healthcare professional students** | | | | | | | | | | | | | | | |
| **Edgar et al. 2021**  [34] | Physiotherapy students (n=65)  Final year of study | Australia | Quantitative questionnaire study with thematic analysis of qualitative free-text responses  Post-intervention survey with comparison to control group | To evaluate the impact of MHFA on confidence to provide MHFA, attitudes towards mental illness and preparedness for practice. | Confidence  Stigma | Standard MHFA course | 13 | Face to face | Compulsory | 71 | Not stated | None | 11 weeks, 8 months | The MHFA intervention group reported higher levels of confidence to provide help (p<0.001) and more positive attitudes towards mental illness (p<0.001) compared to the control group.  Students valued skills gained from MHFA in their clinical practice. | 58 |
| **Forbes et al. 2022**  [21] | Physician assistant students (n=432) from 9 different institutions  All years of study | USA | Quantitative questionnaire study supplemented by qualitative free-text analysis  Post-intervention survey | To assess satisfaction with the MHFA course and confidence in using the skills learned. | Confidence | Standard MHFA course | Not stated | Face to face | Variable, depending on institution | Not stated | Not stated | None | Immediately after | Following MHFA training, students reported high levels of confidence in applying a range of MHFA skills (mean score 4.74 on 5 point scale), and high levels of satisfaction with the course (mean score 4.82 on 5 point scale). | 45 |
| **Ostrowski et al. 2023**  [28] | Occupational therapy students (intervention n=21, control n=21) | USA | Quantitative study  RCT  High risk of bias | To investigate the changes in mental health knowledge, confidence and stigma in occupational therapy students after MHFA training, compared to control group. | Mental health literacy  Confidence  Stigma | MHFA (blended course) | 2 hours online pre-work and 5.5 hour face to face session | Blended | Voluntary | Intervention group 81% female, 19% male. Control group 95% female, 5% male | Intervention group: 24 +/- 2 years. Control group 22+/- 1 year. | Wait list control group | Immediately after | MHFA training was associated with significantly higher scores for mental health knowledge (p=0.026) and confidence compared to the control group, though there were no statistically significant differences in level of stigma (p=0.604). In the intervention group, there were significant improvements from pre to post training. | 65.5  High risk of bias |
| **Wadsworth et al. 2022**  [39] | Paramedic students (n=102)  First and second year | Australia | Quantitative questionnaire study with thematic analysis of qualitative free-text responses  Uncontrolled post-test design | To assess the novelty, relevance and appropriateness of MHFA course for paramedic students, and their individual experiences of participating in training. | Qualitative experiences of participating in the course | MHFA (Blended MHFA for Tertiary students) | Online pre-work and half day face to face session | Blended | Compulsory | 71 | 17-54 | None | Variable, up to 12 months prior to survey | The majority agreed the content was relevant (86%) and appropriate (88%), with 73% agreeing they  would recommend to other university students. Only 44% reported that the course introduced them to new material, indicating significant overlap with their own curriculum or prior knowledge. Thematic analysis identified strengths of the course as perceived increases in mental health literacy and empowerment to act on mental health concerns. A weakness was students perceived the course did not prepare them adequately for clinical practice. | 45 |
| **Mixed student populations** | | | | | | | | | | | | | | | |
| **Bond et al. 2015**  [32] | Medical and nursing students (n=142 medical, n=292 nursing)  All years of study | Australia | Quantitative study  Uncontrolled pre- and post-test | To investigate the impact of MHFA training on MHFA intentions, mental health literacy, confidence in providing help, stigmatising attitudes and course satisfaction | Mental health literacy  Confidence  Intentions  Stigma | Tailored MHFA with elements of youth MHFA | 13 | Face to face (n=261) or online (n=273) | Voluntary | 84.8 | 29.2 (10.59), 17-65 | None | Immediately after | Online and face to face courses for both groups were associated with statistically significant increases in mental health knowledge (p=0.00), confidence (p=0.00), intentions (p=0.00) and social distance (0.00). Findings indicated that online and face to face courses were similarly effective. | 52.5 |
| **Murphy et al. 2023**  [29] | Physiotherapy (n = 73), Physician associate (n = 24),  and OT (n = 29) students | USA | Quantitative study  Uncontrolled pre and post-test design | To investigate the impact of MHFA training on mental health literacy in HCP students (using the MHLS-HC scale) post MHFA training, and post simulation. | Mental health literacy | MHFA | Online pre-work followed by face to face session | Blended | Compulsory | Not stated | Range 21-36 | None | Variable | MHFA training was associated with statistically significant increases in mental health literacy scores for all student disciplines compared to pre-training (p<0.0005). When comparing pre-training to post-simulation, physician associate (p<0.0005) and OT students (p<0.05) showed a statistically significant increase over the longer timeframe, though physiotherapy students did not. | 52.5 |
| **Sibeoni et al. 2023**  [43] | Mixture of different HCP students (n=20) - 12 medical students, 5 pharmacy students, 2 nursing students, 1 physiotherapy student | France | Qualitative study  Semi-structured interviews and thematic analysis of content | To explore the lived experience of French healthcare professional students of MHFA training. | Qualitative experiences of participating in the course | MHFA | 2 days | Face to face | Voluntary | 80 | 19-26 | None | Variable, 1-9 months post | Students reported a personal motivation for taking the course; interest in the topic, to learn and revise Psychiatry, and to develop practical and soft skills for their clinical practice. In their personal experience, students reported a transformative experience and some interventions with friends and family. Students reported that the content and form of the course could be better adapted for their needs. | 48 |

Table 2: Summary of characteristics and findings of included studies.

**Abbreviations:** MHFA = Mental Health First Aid; HCP = Healthcare professional; RCT = randomised controlled trial; SD = Standard deviation; OT = Occupational therapy; MMERSQI = Modified Medical Education Research Study Quality; Instrument; ROB II = Cochrane Risk of Bias II tool (for randomised controlled trials)
