# Supplementary material for: A systematic review of the impact of Mental Health First Aid on medical, nursing and allied healthcare professional students
Source: BJPsych Open. 2026 Sep 1;12(5):e222. doi: 10.1192/bjo.2026.12062 (PMC13748678; doi:10.1192/bjo.2026.12062)
Supplement: Supplementary file 3 [file S2056472426120626sup003.docx]

## February 2026 MHFA systematic review search strategies

Medline (Ovid) searched 04/02/2026

| **#** | **Searches** | **Results** |
| --- | --- | --- |
| 1 | Psychological First Aid/ | 45 |
| 2 | First Aid/ and Mental Health/ | 154 |
| 3 | ("Mental health first aid*" or "MHFA" or MHFAider* or "psychological first aid").ab,kw,ti. | 812 |
| 4 | ("mental health training" or "mental health education" or "mental health literacy").kw,ti. | 1620 |
| 5 | 1 or 2 or 3 or 4 | 2379 |
| 6 | exp Students, Health Occupations/ | 101182 |
| 7 | exp Education, Medical/ | 197987 |
| 8 | exp Education, Nursing/ | 93912 |
| 9 | exp Education, Pharmacy/ | 10612 |
| 10 | Paramedics/ed [Education] | 5 |
| 11 | exp Emergency Medicine/ed [Education] | 6300 |
| 12 | Occupational Therapy/ed [Education] | 1796 |
| 13 | ((medical or health* or nurs*) adj2 (student* or educat* or undergraduate* or school* or training)).ab,kw,ti. | 346774 |
| 14 | 6 or 7 or 8 or 9 or 10 or 11 or 12 or 13 | 563921 |
| 15 | (Medical or physician* or doctor* or nurs* or midwi#e* or "occupational therap*" or physiotherap* or paramedic* or pharmac* or healthcare or "allied health profession*" or "AHP" or "AHPs").ab,kw,ti. | 3989619 |
| 16 | (Student* or pre-registered or pre-registration or preregistered or preregistration or undergraduate* or trainee*).ab,kw,ti. | 497638 |
| 17 | 15 and 16 | 196561 |
| 18 | 14 or 17 | 605825 |
| 19 | 5 and 18 | 913 |

Embase (Ovid) searched 04/02/2026

| **#** | **Searches** | **Results** |
| --- | --- | --- |
| 1 | Psychological First Aid/ | 231 |
| 2 | First Aid/ and Mental Health/ | 401 |
| 3 | ("Mental health first aid*" or "MHFA" or MHFAider* or "psychological first aid").ab,kw,ti. | 946 |
| 4 | ("mental health training" or "mental health education" or "mental health literacy").kw,ti. | 1881 |
| 5 | 1 or 2 or 3 or 4 | 2932 |
| 6 | exp health student/ | 171395 |
| 7 | exp medical education/ | 413661 |
| 8 | exp paramedical education/ | 109766 |
| 9 | ((medical or health* or nurs*) adj2 (student* or educat* or undergraduate* or school* or training)).ab,kw,ti. | 415850 |
| 10 | 6 or 7 or 8 or 9 | 797003 |
| 11 | (Medical or physician* or doctor* or nurs* or midwi#e* or "occupational therap*" or physiotherap* or paramedic* or pharmac* or healthcare or "allied health profession*" or "AHP" or "AHPs").ab,kw,ti. | 5807396 |
| 12 | (Student* or pre-registered or pre-registration or preregistered or preregistration or undergraduate* or trainee*).ab,kw,ti. | 654989 |
| 13 | 11 and 12 | 271914 |
| 14 | 10 or 13 | 871462 |
| 15 | 5 and 14 | 1156 |

PsycInfo (Ovid) searched 04/02/2026

| **#** | **Searches** | **Results** |
| --- | --- | --- |
| 1 | Psychological First Aid/ | 357 |
| 2 | exp mental health literacy/ | 1073 |
| 3 | ("Mental health first aid*" or "MHFA" or MHFAider* or "psychological first aid").ab,ti. | 722 |
| 4 | ("mental health training" or "mental health education" or "mental health literacy").ti. | 1112 |
| 5 | 1 or 2 or 3 or 4 | 2204 |
| 6 | medical students/ | 16600 |
| 7 | nursing students/ | 8294 |
| 8 | exp medical education/ | 29315 |
| 9 | exp nursing education/ | 8876 |
| 10 | ((medical or health* or nurs*) adj2 (student* or educat* or undergraduate* or school* or training)).ab,ti. | 95195 |
| 11 | 6 or 7 or 8 or 9 or 10 | 112477 |
| 12 | (Medical or physician* or doctor* or nurs* or midwi#e* or "occupational therap*" or physiotherap* or paramedic* or pharmac* or healthcare or "allied health profession*" or "AHP" or "AHPs").ab,ti. | 576815 |
| 13 | (Student* or pre-registered or pre-registration or preregistered or preregistration or undergraduate* or trainee*).ab,ti. | 731162 |
| 14 | 12 and 13 | 63602 |
| 15 | 11 or 14 | 135775 |
| 16 | 5 and 15 | 736 |

CINAHL (Ebsco) searched 04/02/2026

| **#** | **Query** | **Results** |
| --- | --- | --- |
| S1 | (MH "Psychological First Aid") | 136 |
| S2 | TI ( "Mental health first aid*" or "MHFA" or MHFAider* or "psychological first aid" ) OR AB ( "Mental health first aid*" or "MHFA" or MHFAider* or "psychological first aid" ) | 468 |
| S3 | TI ("mental health training" or "mental health education" or "mental health literacy") | 891 |
| S4 | S1 OR S2 OR S3 | 1,373 |
| S5 | (MH "Students, Health Occupations+") | 109,213 |
| S6 | (MH "Education, Health Sciences+") | 318,465 |
| S7 | TI ( (medical or health* or nurs*) N1 (student* or educat* or undergraduate* or school* or training) ) OR AB ( (medical or health* or nurs*) N1 (student* or educat* or undergraduate* or school* or training) ) | 185,379 |
| S8 | S5 OR S6 OR S7 | 472,481 |
| S9 | TI ( Medical or physician* or doctor* or nurs* or midwi?e* or "occupational therap*" or physiotherap* or paramedic* or pharmac* or healthcare or "allied health profession*" or "AHP" or "AHPs" ) OR AB ( Medical or physician* or doctor* or nurs* or midwi?e* or "occupational therap*" or physiotherap* or paramedic* or pharmac* or healthcare or "allied health profession*" or "AHP" or "AHPs" ) | 1,660,220 |
| S10 | TI ( Student* or pre-registered or pre-registration or preregistered or preregistration or undergraduate* or trainee* ) OR AB ( Student* or pre-registered or pre-registration or preregistered or preregistration or undergraduate* or trainee* ) | 258,179 |
| S11 | S9 AND S10 | 117,704 |
| S12 | S8 OR S11 | 491,070 |
| S13 | S4 AND S12 | 591 |

ERIC, searched 04/02/2026

("Mental health first aid" OR MHFA OR MHFAider OR "Psychological first aid") AND ((Medical OR physician OR doctor OR nurse OR midwife OR "occupational therapist" OR physiotherapist OR paramedic OR pharmacist OR healthcare OR "allied health profession" OR "AHP") AND (Student OR pre-registered OR undergraduate OR trainee))

4 results

(descriptor:"Medical Students" OR descriptor:"Nursing Students" OR descriptor:"Premedical Students" OR descriptor:"Allied Health Occupations Education" OR descriptor:"Medical Education" OR descriptor:"Medical Schools" OR descriptor:"Nursing Education") AND ("Mental health first aid" OR MHFA OR MHFAider OR "Psychological first aid")

2 results

**Total results**

Before de-duplication: 3402

After de-duplication: 2365

De-duplication using RefWorks reference management software

(No limits or filters used in the database searches)
